# Multi-omics analysis revealed that the protein kinase MoKin1 affected the cellular response to endoplasmic reticulum stress in the rice blast fungus, *Magnaporthe oryzae*

**DOI:** 10.1186/s12864-024-10337-8

**Published:** 2024-05-07

**Authors:** Lianhu Zhang, Yifan Zhang, Yankun Liu, Wenjing Miao, Jingyu Ai, Jingling Li, Song Peng, Songyan Li, Lifang Ye, Rong Zeng, Xugen Shi, Jian Ma, Yachun Lin, Weigang Kuang, Ruqiang Cui

**Affiliations:** 1 College of Agronomy, Jiangxi Agricultural University, Nanchang, 330045 Jiangxi China; 2 Key Laboratory of Crop Physiology, Ecology and Genetic Breeding, Ministry of Education, Jiangxi Agricultural University, Nanchang, 330045 Jiangxi China; 3 College of Bioscience and Bioengineering, Jiangxi Agricultural University, Nanchang, 330045 Jiangxi China

**Keywords:** Mokin1, ER stress, Proteomic, Phosphoproteomic, Transcriptomic

## Abstract

**Background:**

Previous studies have shown that protein kinase MoKin1 played an important role in the growth, conidiation, germination and pathogenicity in rice blast fungus, *Magnaporthe oryzae*. *ΔMokin1* mutant showed significant phenotypic defects and significantly reduced pathogenicity. However, the internal mechanism of how MoKin1 affected the development of physiology and biochemistry remained unclear in *M. oryzae*.

**Result:**

This study adopted a multi-omics approach to comprehensively analyze MoKin1 function, and the results showed that MoKin1 affected the cellular response to endoplasmic reticulum stress (ER stress). Proteomic analysis revealed that the downregulated proteins in *ΔMokin1* mutant were enriched mainly in the response to ER stress triggered by the unfolded protein. Loss of MoKin1 prevented the ER stress signal from reaching the nucleus. Therefore, the phosphorylation of various proteins regulating the transcription of ER stress-related genes and mRNA translation was significantly downregulated. The insensitivity to ER stress led to metabolic disorders, resulting in a significant shortage of carbohydrates and a low energy supply, which also resulted in severe phenotypic defects in *ΔMokin1* mutant. Analysis of MoKin1-interacting proteins indicated that MoKin1 really took participate in the response to ER stress.

**Conclusion:**

Our results showed the important role of protein kinase MoKin1 in regulating cellular response to ER stress, providing a new research direction to reveal the mechanism of MoKin1 affecting pathogenic formation, and to provide theoretical support for the new biological target sites searching and bio-pesticides developing.

**Supplementary Information:**

The online version contains supplementary material available at 10.1186/s12864-024-10337-8.

## Introduction

Rice is the most important food crop and plays a vital role in world food security [1]. However, every year in agricultural production, pests and diseases cause enormous yield losses. Among these diseases, rice blast is the most common disease and seriously affects the yield and quality of rice, so the prevention and control of blast disease are very important for ensuring the agricultural safe production [2]. In agricultural production, it is the most economical and effective means to use disease-resistant varieties to control rice blast disease, but with the population dynamics of the physiological species in the field, disease-resistant varieties often become susceptible varieties, and diseases will break out. Therefore, deepening understanding the mechanism of the fungal pathogenicity formation is an important way to control rice blast epidemics.

In order to clarify the underlying pathogenic mechanism, we first need to understand the infection process and its regulatory pathways. The infection started with the conidium. The conidium that attaches to the rice leaf surface begin to germinate, forming a bud tube, and the tip of the bud tube expanded to form an appressorium. Under the action of glycerol turgor pressure, the bottom of the appressorium formed a penetration peg, which pierced the rice epidermal cells to complete the infection. A few days later, the rice leaves formed disease spots at the infection site, and the pathogen released a large number of conidia, which spread with wind and rain, forming disease cycles and epidemics [3]. The infection process of *M. oryzae* is regulated by a series of genes, which constitute a complex signal transduction process. For example, cAMP and MAPK signaling pathways regulated conidial recognition of the rice leaf epidermis and germination to form the appressorium [4, 5]. In addition, the energy substances stored in the conidium, including glycogen, fat components, and various other nutrients, were transported into appressorium and metabolized into glycerol, accompanied by conidial autophagy and melanin deposition in the appressorial cell wall [6–8]. Eventually, glycerol aggregated to form appressorial pressure. The pressure-sensitive histidine aspartate kinase Sln1 sensed appressorial pressure and recruited the Septin protein and exocyst complex to aggregate at the bottom of the appressorium to form a ring structure [9–11]. Moreover, the protein kinase MoCK2 also formed a ring structure perpendicular to the Septin-Exocyst complex [12, 13]. These dynamic changes were important manifestations of appressorial polar regeneration and regulated the differentiation and formation of penetration peg [14].

The Kin1/PAR-1/MARK family comprises serine/threonine protein kinases. From single-celled *Saccharomyces cerevisiae* strains to mammals, members of this kinase family are highly conserved and share the same domain, including the N-terminal serine/threonine catalytic domain (S_TKc) and the C-terminal regulatory domain composed of approximately 40 amino acids (KA1) [15]. Some members, such as the Rad23, have a UBA domain, which binds to ubiquitin chains and participates in proteasome-regulated proteolysis [16]. Members of Kin1/PAR-1/MARK kinase family have a variety of functions, including cell polarity, microtubule stability, protein stability, extracellular signaling, and cell cycle regulation [17–19]. Previous studies have shown that the protein kinase Kin1 homologus FgKin1 also played an important role in the plant pathogenic fungus *Fusarium graminearum*, as indicated by its involvement in conidiogenesis, pathogenesis, autoinhibition of ascospore germination, and ascospore release [20]. Similarly, the Kin1 homologous protein MoKin1 had a similar function to FgKin1 in *M. oryzae* and played an important role in pathogenicity [20]. However, how MoKin1 participates in physiological and biochemical processes and affects the growth, conidiation and pathogenicity is still unknown. Therefore, analysis of the precise function of MoKin1 has important reference value for understanding the pathogenic formation in *M. oryzae*.

Endoplasmic reticulum stress (ER stress) refers to the excessive accumulation of unfolded or misfolded proteins in the endoplasmic reticulum (ER) and affects the normal function of the ER. ER receptors transmit signals to the nucleus through a series of processes to promote the transcriptional upregulation of specific target genes; these genes encode proteins relating to degrade or reduce unfolded proteins in ER and relieve cell stress, which is also known as the unfolded protein response (UPR). Moderate ER stress can activate a protective mechanism in cells. However, excessive or persistent ER stress can lead to cell death or apoptosis [21]. The monoterpene carvacrol is an effective fungicide that mainly acts on ER and destroys its integrity. However, *Candida albicans* induced ER stress when subjected to the selective pressure of the monoterpene carvacrol and showed resistance through the IRE1-mediated UPR to restore protein folding homeostasis [22]. *Aspergillus fumigatus* also relied on the UPR to maintain survival under stressful conditions, such as through cell wall/membrane homeostasis, hypoxia adaptation, and antifungal resistance [23]. ER stress was related to pathogenicity in plant pathogenic fungi. *Alternaria brassicicola*, a typical necrotic fungus, secreted a large amount of hydrolase and toxins to directly kill host cells during infection, but the UPR mutant of the *hacA* gene could not penetrate healthy leaves of *Arabidopsis thaliana* to cause disease, suggesting that the UPR was necessary for the secretion of sufficient cell wall-degrading enzymes [24]. Disruption of the LHS1 gene in *M. oryzae* triggered the UPR. This mutant obstructed the secretion of effectors, affected infection and reduced pathogenicity [25]. Therefore, an in-depth understanding of ER stress is highly important for understanding the pathogenicity of *M. oryzae*.

In this study, proteomic, phosphoproteomic and transcriptomic methods were adopted to reveal that protein kinase MoKin1 affected the signal transduction of cellular response to ER stress, providing a new research direction for further explaining the role of MoKin1 to regulate pathogenic formation in *M. oryzae*. In addition, the identification of MoKin1 interacting proteins and the screening of available biological targets provided theoretical support for the development of bio-pesticides and the control of rice blast disease.

## Results

### *ΔMokin1* mutant exhibited severe phenotypic defects

**Fig. 1:**
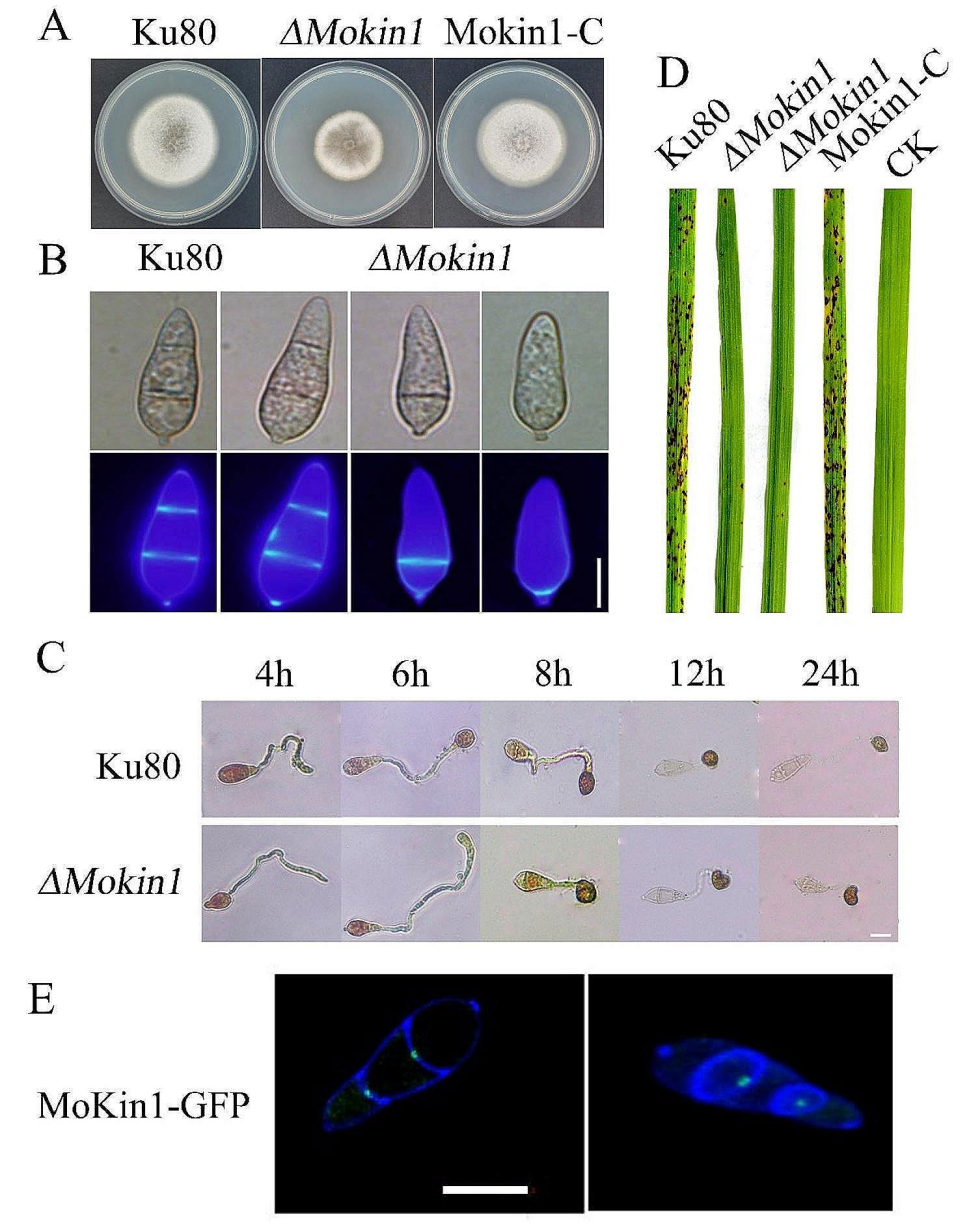
Phenotypic analysis of *ΔMokin1* mutant. (A) Colony morphology of background strains Ku80, *ΔMokin1* mutant and the complementary strains Mokin1-C. (B) Conidial morphology and membrane differentiation in *ΔMokin1* mutant. The septum of the conidium was stained by CFW (Calcofluor white; CFW). (C) Analysis of glycogen mobilization and transformation during conidial germination in *ΔMokin1* mutant. Glycogen staining solution containing 60 mg of KI and 10 mg of I_2_ per milliliter of distilled water. (D) Pathogenicity analysis of *ΔMokin1* mutant. Disease symptoms on rice leaves of 2-week-old seedlings inoculated with conidia suspension. The concentration of conidia in the suspension was about 1 × 10^5^/ml. Typical leaves were photographed 6 days after inoculation. The rice cultivar was CO-39. (E) MoKin1-GFP localization in conidial septal pore. The septum of the conidium was stained by CFW (Calcofluor white; CFW). All bars = 10 μm

The protein kinase MoKin1 played an important role in the rice blast fungus *M. oryzae*. Compared with background strains Ku80, the growth rate of *ΔMokin1* mutant decreased dramatically on starchy yeast media. The growth defects were rescued in the complemented strains Mokin1-C (Fig 1A). The conidial formation of *ΔMokin1* was also approximately half that of Ku80 (Fig S1). Moreover, CFW staining revealed that 70% of the conidia in *ΔMokin1* mutant had two septa and 30% had one or no septa (Fig 1B). The conidia of *ΔMokin1* mutant could form appressoria normally, but a turgor pressure test showed that *ΔMokin1* mutant was more sensitive to hyperosmotic reactions. After treatment with 2 M glycerol for 5 min, approximately 70% of the appressoria in *ΔMokin1* mutant strain collapsed, while approximately 30% of the appressoria in the Ku80 strain collapsed (Fig S2). The measurement of glycogen mobilization during conidial germination also showed that most glycogen were not converted into glycerol in *ΔMokin1* mutant. However, the glycogen in the appressorium of Ku80 was converted into glycerol. The color of the glycogen staining in the appressorium was significantly lighter than that in *ΔMokin1* mutant (Fig 1C). A defect in turgor pressure directly reduced pathogenicity, so a pathogenicity experiment showed that, compared with Ku80, *ΔMokin1* mutant produced fewer disease spots on rice leaves (Fig 1D). In addition, we detected the localization of MoKin1 in *M. oryzae* and found that the fluorescence of MoKin1-GFP was concentrated in the septal pore (Fig 1E). These results demonstrated the important role of the protein kinase MoKin1 in *M. oryzae*.

To determine the precise roles of the MoKin1 regulatory pathways in development of physiology and biochemistry, we adopted multi-omics approach to study MoKin1 in order to gain a new research for MoKin1 in *M. oryzae*.

### Proteomic data in *ΔMokin1* mutant

It is often said in the biological field that proteins are the embodiment of life activity. Therefore, the effect of MoKin1 deficiency on the total protein content of *M. oryzae* was studied via proteomics.

To ensure the smooth implementation of 4D-label-free proteomic quantitative detection, total protein extracts of the Ku80 and *ΔMokin1* mutants were analyzed via SDS‒PAGE. The results showed that the quality of the total protein extracted from the 6 samples was very high, which met the requirements of subsequent experiments (Fig. S3). These proteins were then subjected to enzyme digestion and LC‒MS/MS analysis. MaxQuant software was used to combine and retrieve MS raw data for each sample for identification and quantitative analysis (Table. S1).

**Fig. 2:**
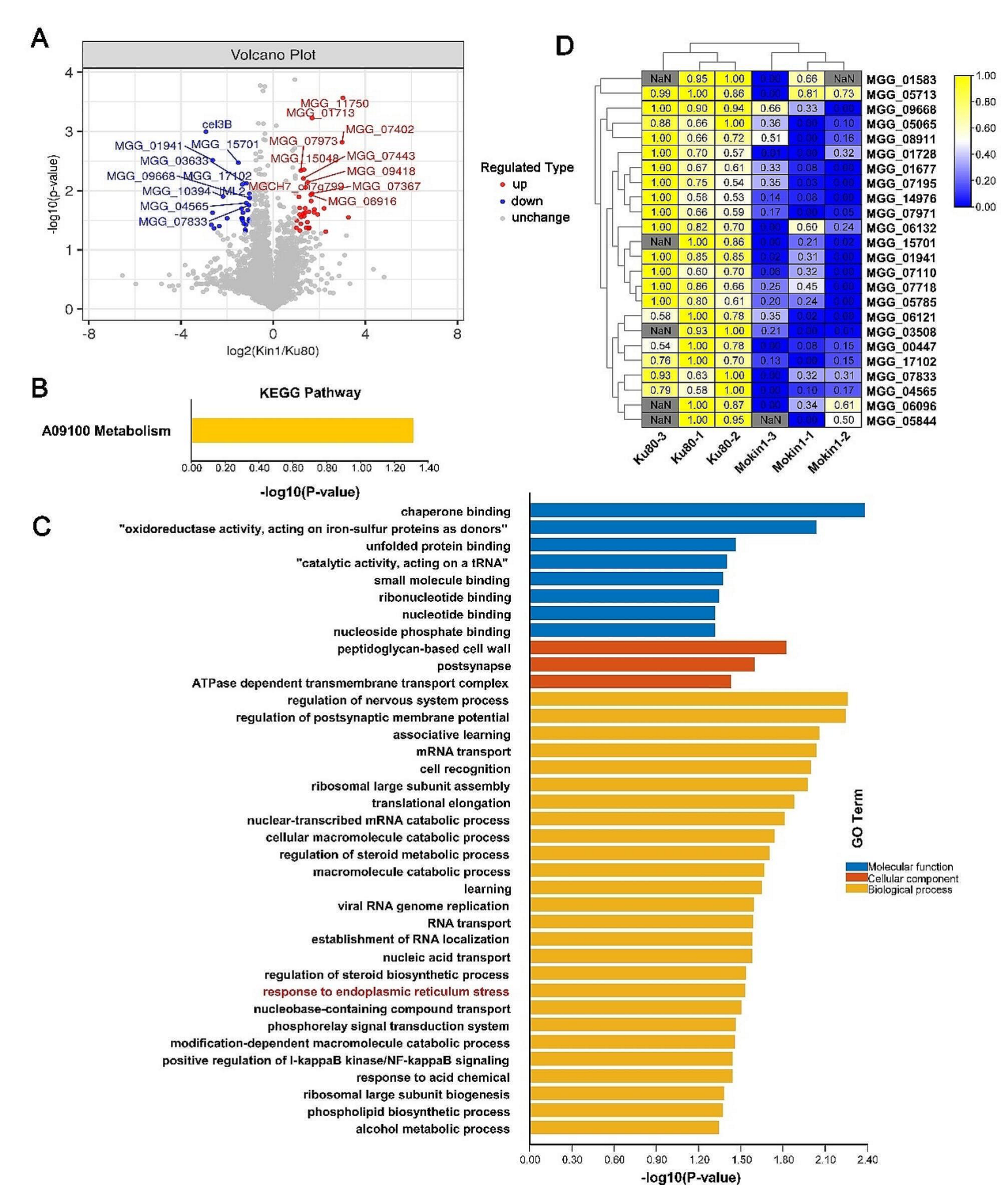
Differential expression protein (DEP) analysis in *ΔMokin1* mutant. (A) Distribution of the total identified protein is shown by the volcano plot. The *x*-axis shows various fold change groups and the *y*-axis shows the *p*-value. (B) Down-regulated DEPs in the KEGG pathway. (C) Down-regulated DEPs in the GO terms. (D) Heat map analysis of protein expression levels involved in endoplasmic reticulum stress response

In this study, proteins with *P* values < 0.05 and absolute fold change values > 2 were considered as the significantly differentially expressed proteins (DEPs). The volcano plot revealed 62 DEPs between *ΔMokin1* mutant and Ku80. Among them, 34 proteins were upregulated, and 28 proteins were downregulated (Fig 2A and Table. S1). Hierarchical clustering also revealed similar expression patterns of these proteins in the different groups (Fig S4).

### KEGG enrichment of DEPs in *ΔMokin1* mutant

To better understand the function of the protein kinase MoKin1, we first analyzed DEPs through Kyoto Encyclopedia of Genes and Genomes (KEGG) analysis using TBtools [26]. KEGG enrichment revealed that the downregulated DEPs were significantly involved in only one pathway (A09100 Metabolism), which included eight proteins (Fig 2B and Table.S1).

MGG_01728 encodes methylenetetrahydrofolate reductase 1. Methylene tetrahydrofolate reductase catalyzes the reduction of 5, 10-methylene tetrahydrofolate (CH2-THF) to 5-methyltetrahydrofolate (CH3-THF), which is necessary for L-methionine biosynthesis. Deletion of MGG_01728 in *M. oryzae* produced severe phenotypic defects and inhibited methionine synthesis and pathogenicity [27]. In addition, O-acetylhomoserine (OAH) is another important precursor of L-methionine synthesis. O-acetylhomoserinelyase (encoded by MGG_07195) can promote the reaction of o-acetylhomoserine (OAH) with methylmercaptan to produce L-methionine, which is involved in the metabolism of methionine [28].

Levanase, encoded by MGG_05785, and beta-glucosidase cel3A, encoded by MGG_03508, are N-glycosylated proteins related to the ER quality control (ERQC) system and play important roles in the infection process of *M. oryzae* [29]. Moreover, MGG_05785, MGG_03508 and MGG_05844 (MGG_05844 encods mannan Endo-1,4-beta-mannosidase) are also *Magnaporthe* effector proteins (MEPs) that are closely related to pathogenicity in *M. oryzae* [30].

The remaining three proteins, hydroxytetrahydrobiopterin dehydratase encoded by MGG_06132, may participate in the process of aromatic amino acid metabolism. Phosphatidylethanolamine N-methyltransferase encoded by MGG_07110 and ethanolamine kinase encoded by MGG_05713, participate in the metabolism of ethanolamine, which may be involved in the secondary metabolism of *M. oryzae*.

From the results above, it can be seen that the loss of MoKin1 has a serious impact on some metabolism of *M. oryzae*, such as amino acid biosynthesis, endoplasmic network homeostasis and secreted protein synthesis, and secondary metabolic processes.

### GO enrichment of DEPs in *ΔMokin1* mutant

To further analyze the function of the DEPs, we adopted the strategy of Gene Ontology (GO) analysis. According to the classification analysis, the DEPs were involved mainly in molecular functions (MF), cell components (CC) and biological processes (BP). In the MF category, DEPs primarily function as molecular chaperones, binding nucleic acids and unfolded proteins. With regard to the CC category, DEPs were mainly involved in the cell wall and membrane. In the BP category, DEPs were involved in various biological processes, such as RNA metabolism and stress response (Fig 2C and Table. S1).

Of these GO terms, we were most interested in the response to ER stress (GO:0034976) (Table.S1). GO enrichment indicated that the loss of MoKin1 led to a decreased response to ER stress. The metabolic intensity relating to ER stress, such as unfolded protein binding (GO:0051082), cellular macromolecular catabolism (GO:0044265), nuclear transcription mRNA catabolism process (GO:0000956), and ribosome large subunit assembly (GO:0000027), also decreased (Fig 2D and Table S1).

The results of GO enrichment of down-regulated DEPs indicated that the deletion of protein kinase MoKin1 disrupted the process of cell response to ER stress, resulting in ER function disorder, then, affecting some protein posttranslational modification.

### Phosphoproteomic data in *ΔMokin1* mutant

**Fig. 3:**
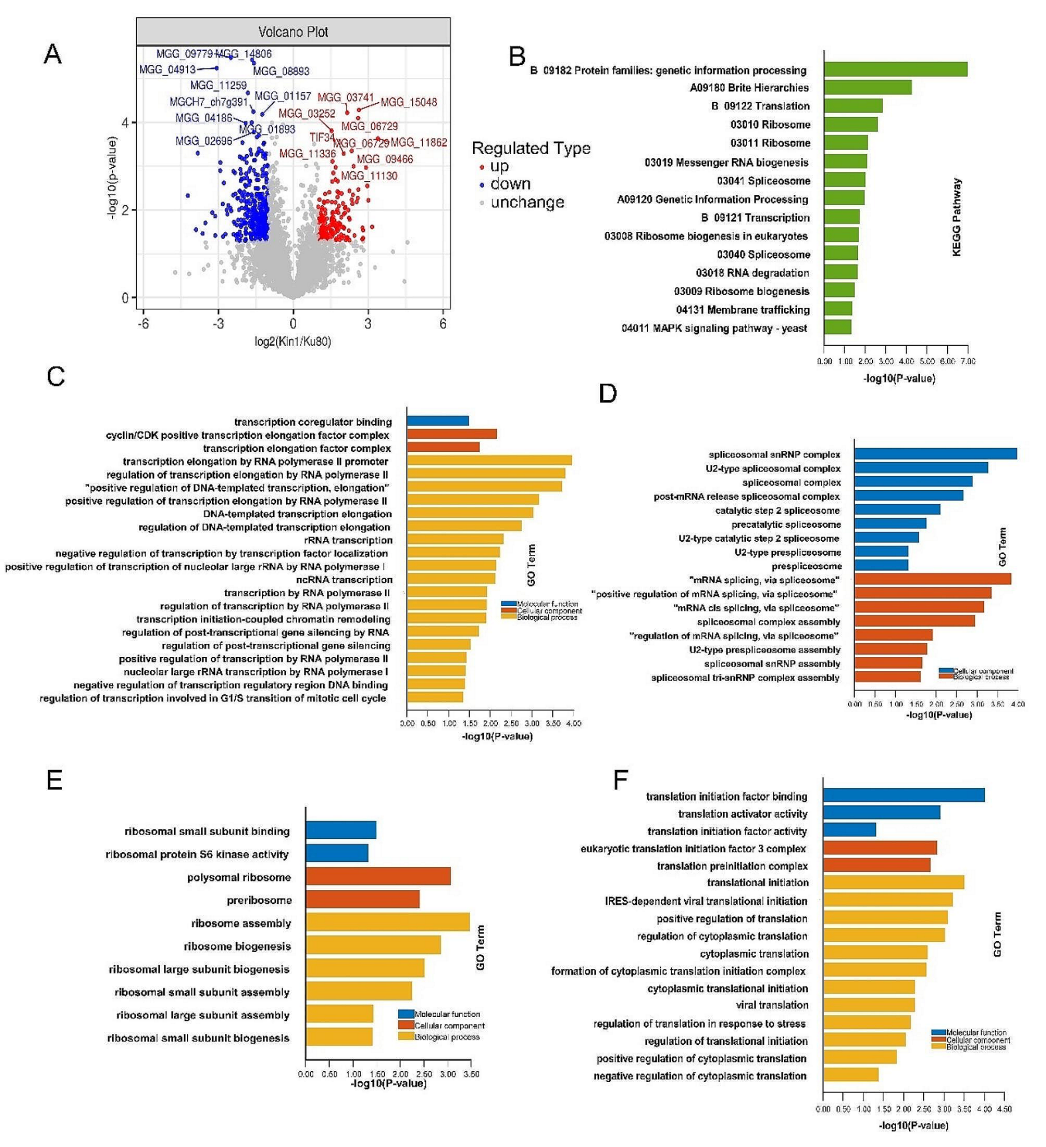
Analysis of phosphoproteins with differential abundance in *ΔMokin1* mutant. (A) The volcano plot showed the phosphoproteins with differential abundance. The *x*-axis shows various fold change groups and the *y*-axis shows the *p*-value. Red color indicated up-regulated and blue color indicated down-regulated. (B) KEGG enrichment of down-regulated phosphoproteins. (C) GO terms of down-regulated phosphoproteins involving in gene transcription. (D) GO terms of down-regulated phosphoproteins involving in spliceosome complex. (E) GO terms of down-regulated phosphoproteins involving in assembly of ribosome. (F) GO terms of down-regulated phosphoproteins involving in protein translation

MoKin1 is a protein kinase, so loss of MoKin1 must result in changes in protein phosphorylation in *M. oryzae* cells. To better understand the changes in phosphorylation events after the deletion of Mokin1, we further researched Ku80 and *ΔMokin1* by phosphoproteomic (Fig S5 and S6). Phosphoproteomic sequencing data showed 3636 phosphoproteins and 12,304 phosphopeptides were identified. Among these, 2647 phosphoproteins and 6972 phosphopeptides were quantified. What’s more, 8,414 phosphosites were also quantified. Among the 8,414 quantified phosphorylation sites, 6673 (79.3%) were phosphoserine serine, 1700 (20.21%) were threonine residues, and 41 (0.49%) were tyrosine residues. Most phosphopeptides are monophosphorylated or diphosphorylated (Fig S7 and Table.S2). The criteria for the identification of phosphoproteins with differential abundance were consistent with the previous criteria for the identification of DEPs. Compared with Ku80, 515 phosphoproteins in *ΔMokin1* mutant were different abundances; 158 were upregulated, and 357 were downregulated (Fig 3A and Table S2).

### Phosphoproteomic analysis in *ΔMokin1* mutant

GO and KEGG methods were used to analyze the phosphoproteomic data to reveal the cellular biochemical processes mediated by these phosphorylation sites. KEGG enrichment analysis revealed that the down-regulated phosphorylated proteins were involved in membrane trafficking, gene transcription, protein translation and kinase signal transduction (Fig 3B and Table.S2). In addition, the GO enrichment results showed that phosphorylation events affected gene transcription and protein translation.

During the process of gene transcription, proteins whose phosphorylation changed mainly affected the elongation process of transcription, such as the transcription elongation factor complex (GO:0008023), transcription elongation by the RNA polymerase II promoter (GO:0006368), and DNA-templated transcription elongation (GO:0006354) (Fig 3C and Table.S2). In addition, changes in protein phosphorylation in *ΔMokin1* mutant affected RNA splicing complex function, including spliceosomal snRNP assembly (GO:0000387) and spliceosomal complex assembly (GO:0000245) (Fig 3D and Table.S2).

A reduction in transcriptional elongation activity directly decreased the amount of primitive RNA in the nucleus. The abnormal assembly of the splicing complex prevents mRNA editing. Both the production and maturity of the mRNAs were already problematic, so the subsequent protein translation process must be impaired in *ΔMokin1* mutant. Our results showed that the biogenesis and assembly of ribosomes, such as ribosome assembly (GO:0042255) and ribosome biogenesis (GO:0042254), were significantly inhibited in *ΔMokin1* mutant (Fig 3E and Table.S2). The corresponding protein translation initiation stage was obviously disordered, and some GO terms were enriched in this stage, including translation factor binding (GO:0031369), eukaryotic translation initiation factor 3 complex (GO:0005852), and translational initiation (GO:0006413) (Fig 3F and Table.S2).

Proteomic analysis showed that the loss of Mokin1 inhibited the cellular response to ER stress, and the nucleus was unable to receive the signals, so the transcription and translation of a series of genes involving in response to ER stress could not be activated. Therefore, the phosphorylation levels of transcription factors and proteins taken participated in transcription and translation were also decreased in*ΔMokin1* mutant.

### Transcriptome information in *ΔMokin1* mutant

The details of the transcriptome data were shown in Table.S3. The sequencing data of the 6 samples ranged from 4.5 Gb to 6.9 Gb. After filtering out low-quality reads and adaptor sequences, 4.5 Gb to 6.8 Gb of high-quality clean reads remained. The Q20 of the clean reads ranged from 97.94 to 98.1%, and the Q30 ranged from 93.74 to 94.21%. In addition, the average GC content was approximately 56.44%, meeting the sequencing requirements (Table.S3). We counted the effective reads compared to the *M. oryzae* genome. More than 92.2% of the readers could map to the genome, and more than 98% of the mapped readers were unique (Table.S3). Principal component analysis (PCA) revealed that the two groups of samples were clearly separated, and the biological replicates within each group were clustered together. The results showed that the difference between the Ku80 and *ΔMokin1* mutants was 71.962% according to PC1, and the difference between the samples was mainly 20.901% according to PC2. The heatmap results showed that the correlation analysis of gene expression levels among the samples showed that the samples had high consistency in one group, which ensured the reliability of the subsequent analyses (Fig S8). The transcriptome data also revealed 3158 new genes and 15,983 annotated genes (Table.S3).

**Fig. 4:**
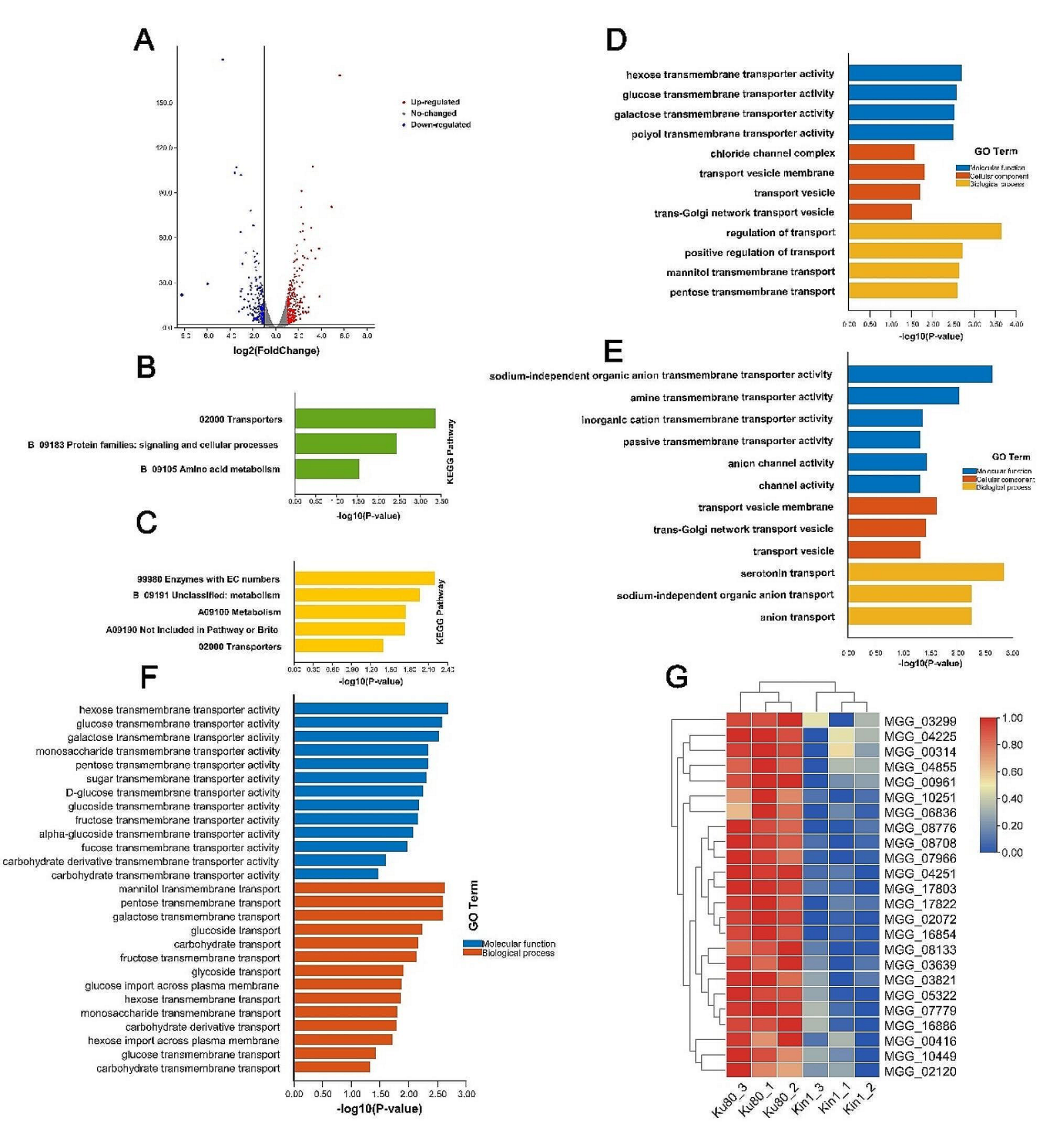
Differential expression genes (DEGs) analysis in *ΔMokin1* mutant. (A) The volcano plot showed the differentially expressed genes (DEGs) in the *ΔMokin1* mutant compared with background strain Ku80. The blue points show the downregulated expression of genes in *ΔMokin1* mutant. The red points show the upregulated expression of genes in *ΔMokin1* mutant. (B) KEGG enrichment of down-regulated genes in *ΔMokin1* mutant; (C) KEGG enrichment of up-regulated genes in *ΔMokin1* mutant. (D) GO enrichment of down-regulated genes in *ΔMokin1* mutant; (E) GO enrichment of up-regulated genes in *ΔMokin1* mutant. (F) GO entries of down-regulated genes enrichment in carbohydrate transport in *ΔMokin1* mutant. (G) Heat maps showed changes in the expression of carbohydrate transport-related genes in *ΔMokin1* mutant compared with Ku80

The criteria for selecting differentially expressed genes (DEGs) were the same as those used in proteomics and phosphorylomics. A volcano plot was constructed, and showed that the expression levels of 593 genes in *ΔMokin1* mutant were significantly different; 344 genes were upregulated, and 249 genes were downregulated compared with Ku80 (Fig 4A and Table.S3).

### Transcriptome analysis in *ΔMokin1* mutant

In general, GO and KEGG analyses were used to analyze the transcriptome data. KEGG pathway analysis revealed that both downregulated and upregulated genes were enriched in transporters (02000 transporters), which was consistent with MoKin1 septal pore localization (Figs. 1E and 4B and C and Table.S4).

GO enrichment analysis also revealed enrichment of downregulated and upregulated genes in the transporter terms (Fig 4D and E and Table.S4). Further analysis revealed that GO terms related to carbohydrate transport were more numerous in down-regulated genes than in up-regulated genes. There were only four GO terms, sucrose transport (GO:0015770), trehalose transport (GO:0015771), disaccharide transport (GO:0015766), and oligosaccharide transport (GO:0015772) in up-regulated genes (Table.S4). However, there were up to 27 GO terms in down-regulated genes, including carbohydrate transport (GO:0008643), carbohydrate transmembrane transport (GO:0034219), glucose transmembrane transport (GO:1,904,659), and fructose transmembrane transport (GO:0015755) (Fig 4F and G and Table.S4). These results suggested that MoKin1 deletion severely inhibited carbohydrate transport and led to a decrease in intracellular energy metabolism.

The cellular response to ER stress is an extremely energy-intensive process, and the transport of carbohydrates in *ΔMokin1* mutants was obstructed, blocking the energy supply and inhibiting the cellular response to ER stress.

### Interacting proteins of Mokin1

Proteomic, phosphoproteomic and transcriptomic data revealed that MoKin1 deletion inhibited the cellular response to ER stress, thereby disrupting normal metabolism and causing phenotypic defects in *ΔMokin1* mutant. However, how MoKin1 regulating the cellular response to ER stress has not been reported in filamentous fungi. To solve this problem, the key point was to identify the interacting proteins of MoKin1.

**Fig. 5:**
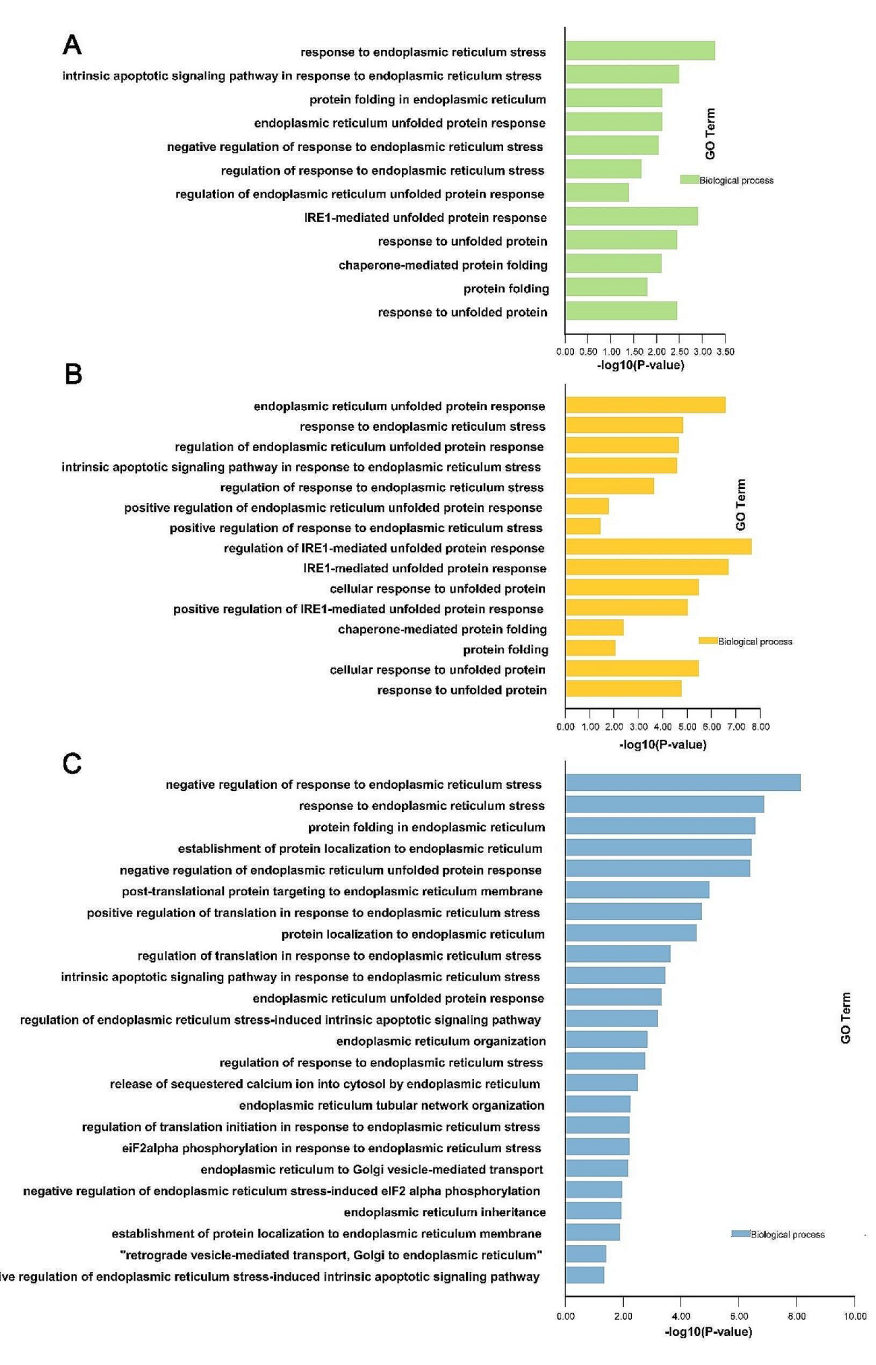
GO enrichment of MoKin1 physical interacting protein (A), genetic interacting protein (B) and putative substrates obtained from MoKin1-pull down (C) in response to ER stress

In this study, homologous proteins of yeast KIN1/KIN2 interacting proteins were pursued in *M. oryzae* by alignment. These results were showed in Table.S5. We conducted a preliminary analysis of these interacting proteins, and the results were presented in Table.S6. Among these results, some proteins physical interaction or genetic interaction with MoKin1 were enriched in response to ER stress, including the ER unfolded protein response (GO:0030968) and regulation of response to ER stress (GO:1,905,897) (Fig 5A, B and Table.S6, Sheet9). In addition, through a MoKin1 pull-down experiment, 837 proteins may interact with MoKin1. Some of these proteins were also enriched in response to ER stress (GO:0034976) (Fig 5C and Table.S7).

Further exploration of the function of MoKin1 interacting proteins in subsequent experiments will help to understand the role of MoKin1 in cell response to ER stress.

## Discussion

The protein kinase MoKin1 played an important role in rice blast fungus. Our research showed that MoKin1 regulated mycelial growth, conidiation, appressorial morphology and pathogenicity. *ΔMokin1* mutant exhibited obvious phenotypic defects. Therefore, to better understand the regulatory network of MoKin1, we adopted multiple omics methods to conduct a comprehensive analysis of MoKin1 to elucidate the pathogenic formation in *M. oryzae*.

**Fig. 6:**
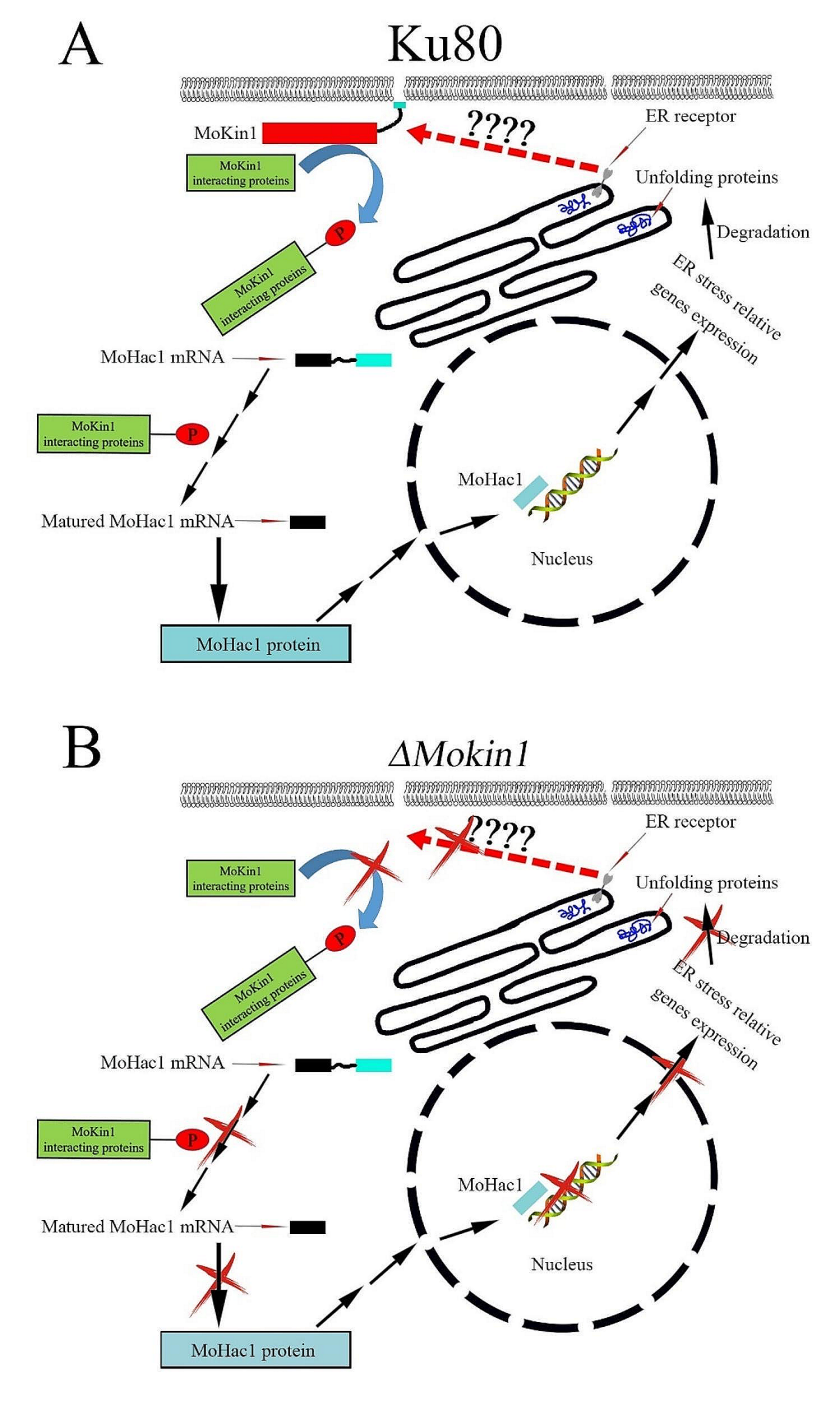
A hypothesized signaling pathway for cell response to ER stress in *M. oryzae*, based on the study of cell response to ER stress in *S. cerevisiae*. When ER is stress, the signal is transmitted to the protein kinase MoKin1, which phosphorylates and activates MoKin1. The activated MoKin1 then acts on downstream interacting proteins, affecting the editing of MoHAC1 mRNA and protein translation. MoHAC1 protein enters the nucleus and regulates gene expression in response to endoplasmic reticulum stress (A). But in *ΔMokin1* mutant, the signal pathway is interrupted (B)

Through proteomic, phosphoproteomic and transcriptomic analysis, we revealed that MoKin1 affected the cellular response to ER stress. In *S. cerevisiae*, after recognizing the signal of unfolded protein accumulation, the RNase activity of bi-functional Ire1 localized to the ER membrane could shear the intron of HAC1 mRNA, promoting the maturation of HAC1 mRNA for translation [31]. During the editing process of HAC1 mRNA, KIN1/KIN2 kinase phosphorylated Pal2, and Pal2 bind to the 3’-UTR of HAC1 mRNA, thus targeting HAC1 mRNA to the correct site to complete the editing process and participating in response to ER stress [32]. Based on the study of cell response to ER stress in *S. cerevisiae*, we could also construct a hypothesized signaling pathway for cell response to ER stress in *M. oryzae*. During ER stress, the signal was transmitted to the protein kinase MoKin1, which phosphorylated MoKin1 and activated MoKin1. Then activated MoKin1 acted on downstream interacting proteins, affecting the editing of MoHAC1 mRNA and translating MoHAC1 protein. MoHAC1 protein entered into the nucleus and regulated the expression of a series of genes to relieve ER stress. However, in *ΔMokin1* mutant, the signal was interrupted due to the absence of MoKin1, and the cells could not respond to ER stress, resulting in metabolic disorders, gene expression and protein translation disorders, and energy transport blocking, resulting in serious phenotypic defects in *ΔMokin1* mutant (Fig 6).

Many proteins were involved in the process of ER stress affecting the pathogenicity formation in *M. oryzae*. The ubiquitin ligase MoHrd1 and the transmembrane protein MoDer1 participated in ubiquitination and transport of the folding-defective proteins, and their mutants induced ER stress, which affected the appressorium development and pathogenicity formation [33]. Some mutants of proteins localizing in the ER, such as MoSCS2, MoSpf1, MoERR1 and MoLHS1, could also cause ER stress and reduced the pathogenicity. ER stress was also involved in the regulation of signal transduction [25, 34–36]. For example, in *S. cerevisiae*, the ER and cell wall were co-ordinated by UPR and CWI signaling pathways to protect cells from the effects of some stressors [37]. ER stress could activate the CWI pathway by increasing MoMps1 phosphorylation level in *M. oryzae*, and the integrity of the CWI pathway was also crucial for UPR activation [38]. At the same time, autophagy protein MoAtg1 could activate the CWI pathway by phosphoylation of MAP kinase kinase MoMkk1, which revealed the synergistic effect of autophagy, ER stress and CWI pathway in pathogenicity formation of *M. oryzae* [39]. These results suggested that ER stress plays an important role in the pathogenesis of *M. oryzae*.

Therefore, in the following studies, we will focus on the study of ER stress, reveal the signaling pathway of MoKin1 regulation of cellular response to ER stress, and provide theoretical support and research direction for the elucidation of the pathogenic formation in *M. oryzae*.

## Materials and methods

### Fungal strains and growth conditions

All the strains used in this study (the background strains Ku80 and *ΔMokin1*) were stored on dry sterile filter paper. The growth phenotype was tested on starchy yeast media (SYM: 0.2% yeast extract, 1% starch, 0.3% sucrose, 1.5% agar). The mycelia used in the experiment were inoculated in complete medium (CM: 0.6% yeast extract, 0.6% casein hydrolysate, 1% sucrose, 1.5% agar) and cultured at 180 rpm at 25 °C. After 4 to 6 days of culture, the cells were filtered, collected, pressed dry, and stored in liquid nitrogen.

### Phenotypic analysis

Ku80, *ΔMokin1* mutant and the complementary strains Mokin1-C were all cultured on starch yeast medium (SYM: 0.2% yeast extract, 1% starch, 0.3% sucrose, 1.5% agar) at 25 °C for ten days. The growth rate of these pathogens was observed and compared by the diameter of the colony.

In order to produce conidia, Ku80, *ΔMokin1* mutant and Mokin1-C were cultured on rice bran medium (1.5% rice bran, 1.5% agar) at 25℃, with alternating dark light for 24 h. After the conidia were collected, the spore suspension was adjusted to the appropriate concentration, and the spore morphology and subcellular localization of MoKin1-GFP were observed. Meanwhile, the conidia were stained with CFW (Calcofluor white: 0.5 ml of 1 mg/ml of calcofluor stock solution was mixed well with 24.5 ml of 0.1 mol/L of Tris-HCl buffer PH 8.5. It is necessary to avoid light during preparation and use.).

Conidia were incubated on hydrophobic microscope cover glasses (Fisherbrand) at 25 °C in the dark to detect the germination and appressorial formation. The glycogen staining solution contains 60 mg of potassium iodide and 10 mg of potassium iodide in per ml of distilled water. After glycogen transformation and decomposition, the color of the conidia or appressoria under the microscope became lighter.

Plant infection assays were performed on rice leaves. The rice cultivar used for infection assays was CO39. In short, the conidial suspensions (1 × 10^5^ conidia/ml in sterile water with 0.02% Tween 20) were sprayed on the rice leaves of 2-week old seedlings. These leaves were incubated in the dark for 24 h and transferred into constant light and incubated for 5 days to assess pathogenicity.

### RNA extraction and RNA sequencing

Total mycelial RNA was extracted using a Sigma‒Aldrich Trading Co. (Shanghai, China) Plant/Fungal RNA Purification Kit. We used an Implen (Germany) P330 Ultra-Micro spectrophotometer to evaluate the integrity and quantity of the RNA. High-integrity RNA was prepared from three biological replicates and used in the preparation of independent libraries. High-throughput sequencing was performed on Illumina HiSeq 2000 machines from Igenebook Biotechnology Co., Ltd. (Wuhan, China).

### Analysis of transcriptome data

The mycelia of blast fungi were collected from 6 samples belonging to two groups, namely Ku80 and Kin1. The Illumina Hi-Seq 2000 platform was used for sequencing. Low-quality sequencing data and adaptor sequences were removed with Trimmomatic to obtain clean reads, and the quality of these reads was evaluated based on Q20, Q30, and GC content [40]. These clean data were aligned to the *M. oryzae* reference genome using HISAT2 software [41]. After obtaining valid reads, we used featureCounts (version: v1.6.0) to calculate the number of reads matching with genes, based on *M. oryzae* genomic annotation file [42]. Since the sequencing amount of each sample is different, the number of same gene reads needed to be standardized in order to compare their expression difference horizontally among different samples. The standardized method used here is FPKM (Fragments per kilobase of exon per million reads mapped) [43]. The FPKM values can be used for a series of subsequent analyses. Principal component analysis (PCA) studies the relationship of main components between samples. Horizontal axis PC1 mainly indicates the difference between the sample groups, that is, the experimental group and the control group. A larger PC1 value indicates that the treatment used in the experiment has a greater influence on the sample. The vertical axis PC2 mainly indicates the repeatability of data between samples in the group. The smaller the PC2 value, the better the reliability of sequencing data of each sample [44]. According to the gene expression levels of each sample, pearson correlation coefficient was used to calculate the correlation between the samples and made a heatmap. The higher the correlation, the darker the color [45]. The results of PCA and heatmap were shown in Fig S8. Differential expression analysis was performed using the R package edgeR, with genes having an FDR value < 0.05 and an absolute value of FoldChange > 2 indicating significantly differentially expressed genes (DEGs) [46]. These DEGs were listed in Table.S3. GO and KEGG enrichment analysis were performed based on these DEGs.

### Protein extraction and digestion

SDT (4% SDS, 100 mM Tris-HCl, pH 7.6) buffer was used for sample lysis and protein extraction. The amount of protein was quantified with a BCA protein assay kit (Bio-Rad, USA). Twenty micrograms of protein from each sample was mixed with 5X loading buffer and boiled for 5 min. The proteins were separated on a 4-20% SDS‒PAGE gel (constant voltage 180 V, 45 min). Protein bands were visualized by Coomassie blue R-250 staining. Protein digestion by trypsin was performed according to the filter-aided sample preparation (FASP) procedure described by Matthias Mann [47]. The digested peptides of each sample were desalted on C18 cartridges (Empore™ SPE Cartridges C18 (standard density), bed I.D. 7 mm, volume 3 ml; Sigma), concentrated by vacuum centrifugation and reconstituted in 40 µl of 0.1% (v/v) formic acid.

### IMAC enrichment method

The enrichment of phosphopeptides was carried out using a HighSelectTM Fe-NTA Phosphopeptide Enrichment Kit according to the manufacturer’s instructions (Thermo Scientific). After lyophilization, the phosphopeptide peptides were resuspended in 20 µL of loading buffer (0.1% formic acid).

### LC–MS/MS analysis

LC‒MS/MS analysis was performed on a timsTOF Pro mass spectrometer (Bruker) coupled to a NanoElute (Bruker Daltonics) for 60 min. The peptides were loaded on a C18 reversed-phase analytical column (homemade, 25 cm long, 75 μm inner diameter, 1.9 μm; C18) in buffer A (0.1% formic acid) and separated with a linear gradient of buffer B (84% acetonitrile and 0.1% formic acid) at a flow rate of 300 nl/min. The mass spectrometer was operated in positive ion mode. The mass spectrometer collected ion mobility MS spectra over a mass range of m/z 100–1700 and 1/k0 of 0.6 to 1.6 and then performed 10 cycles of PASEF MS/MS with a target intensity of 1.5k and a threshold of 2500. Active exclusion was enabled with a release time of 0.4 min.

### Identification and quantitation of proteins

The MS raw data for each sample were combined and searched using MaxQuant software for identification and quantitation analysis [48]. The reference genome sequence of *M. oryzae* 70−15 was downloaded from UniProt, a public database (http://www.uniprot.org/).

### Electronic supplementary material

Below is the link to the electronic supplementary material.

Supplementary Material 1

Supplementary Material 2

Supplementary Material 3

Supplementary Material 4

Supplementary Material 5

Supplementary Material 6

Supplementary Material 7

Supplementary Material 8

## Data Availability

The MoKin1 RNA-Seq data described in this paper have been submitted to the Genome Sequence Archive at the National Genomics Data Center (NGDC) (https://ngdc.cncb.ac.cn/) under accession number CRA013045.The MoKin1 proteome and phosphorylation group data reported in this paper have been deposited in OMIX, China National Center for Bioinformation/Beijing Institute of Genomics, Chinese Academy of Sciences (https://ngdc.cncb.ac.cn/omix: accession no. OMIX005090).
