# Supplementary material for: Multi-omics analysis revealed that the protein kinase MoKin1 affected the cellular response to endoplasmic reticulum stress in the rice blast fungus, Magnaporthe oryzae
Source: BMC Genomics. 2024 May 7;25:449. doi: 10.1186/s12864-024-10337-8 (PMC11077741; doi:10.1186/s12864-024-10337-8)
Supplement: Supplementary file 8 — Supplementary Material 8 [file 12864_2024_10337_MOESM8_ESM.docx]

Supplementary Information


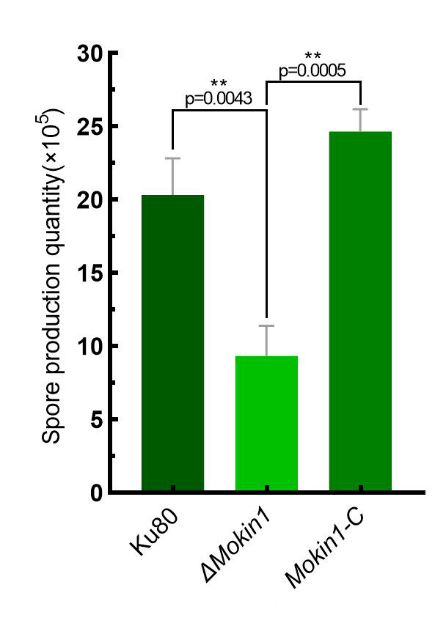


Fig.S1 Conidial formation of background strains Ku80, *ΔMokin1* mutant and the complementary strains Mokin1-C.


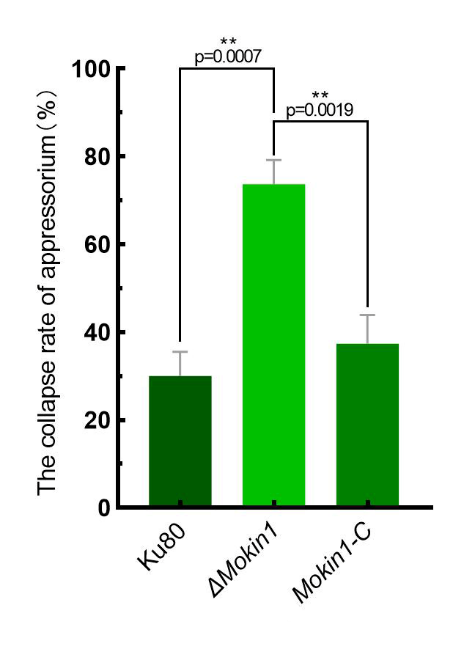


Fig.S2 Appressorial turgor pressure test in background strains Ku80, *ΔMokin1* mutant and the complementary strains Mokin1-C.


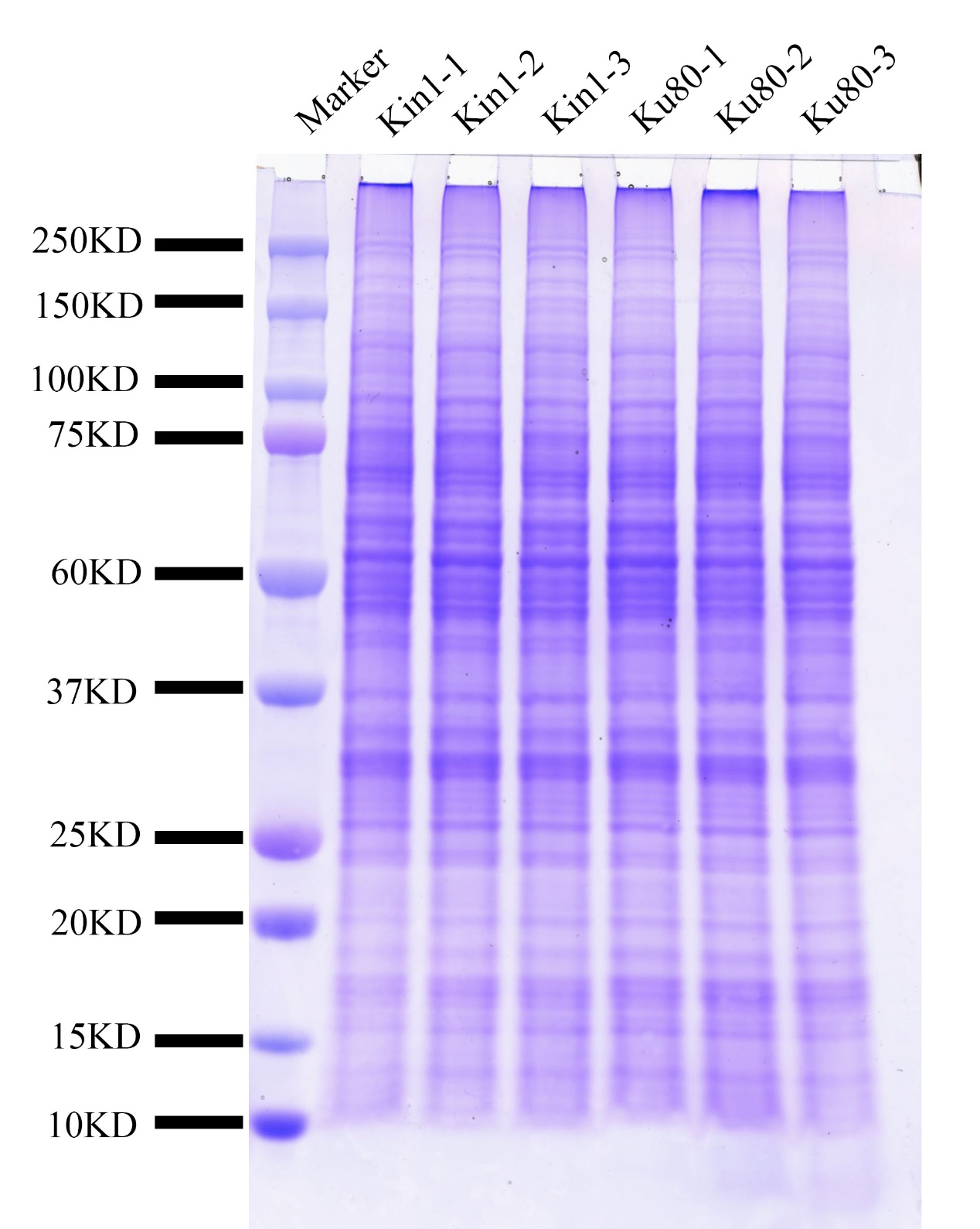


Fig.S3 The total protein of *M. oryzae* for proteome analysis was extracted by TCA/ acetone precipitation +SDT cleavage method, and then carried on sodium dodecyl sulfate polyacrylamide gel electrophoresis (SDS-PAGE).


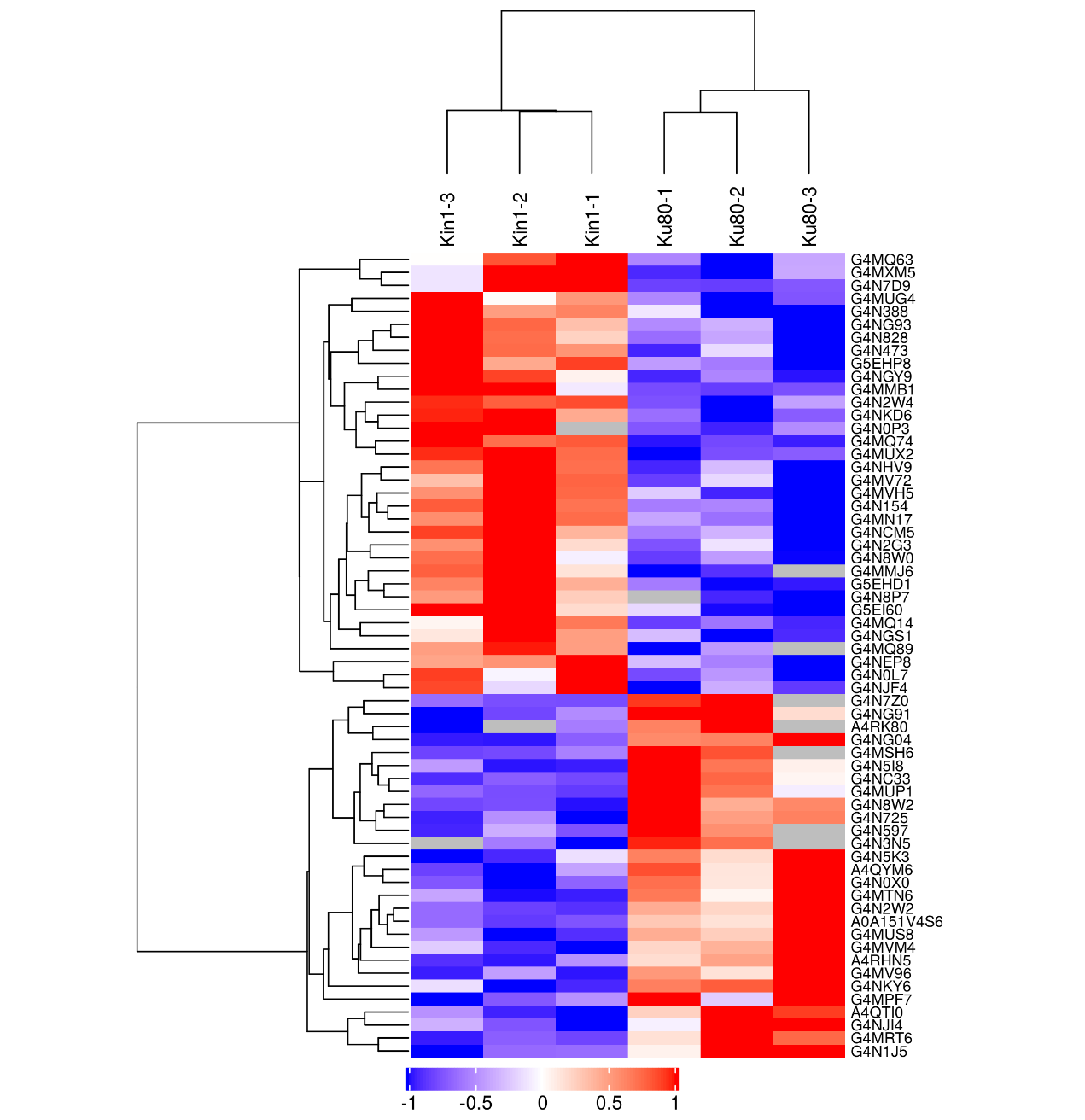


Fig.S4 Hierarchical clustering was used to analyze the DEPs in compared group *ΔMokin1*_vs_Ku80. Heat map showed the similar expression patterns of different samples. Red meant a high relative expression level, whereas blue meant a low relative expression level.


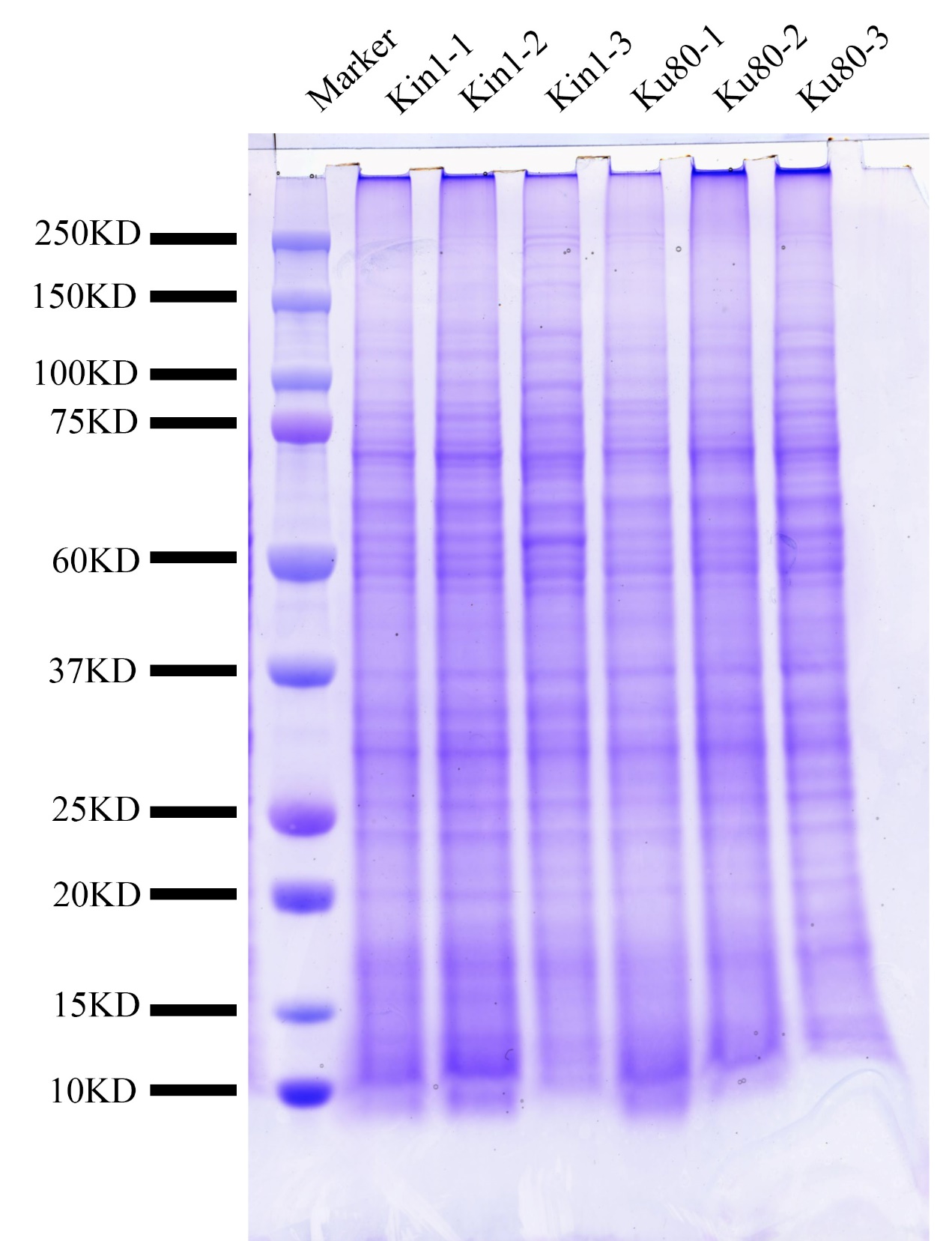


Fig.S5 The total protein of *M. oryzae* for phosphorylomics analysis was extracted by TCA/ acetone precipitation + UA cleavage method, and then subjected to sodium dodecyl sulfate polyacrylamide gel electrophoresis (SDS-PAGE).


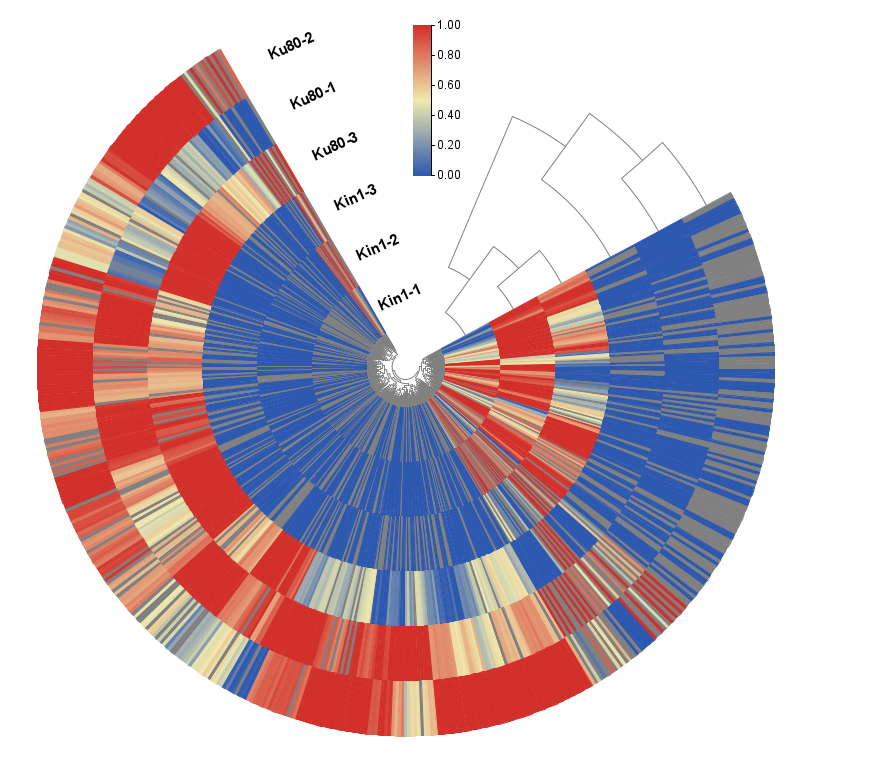


Fig.S6 Hierarchical clustering was used to analyze the DPSs in compared group *ΔMokin1*_vs_Ku80. Heat map shows the similar expression patterns of different samples. Red mean a high relative expression level, whereas blue mean a low relative expression level.


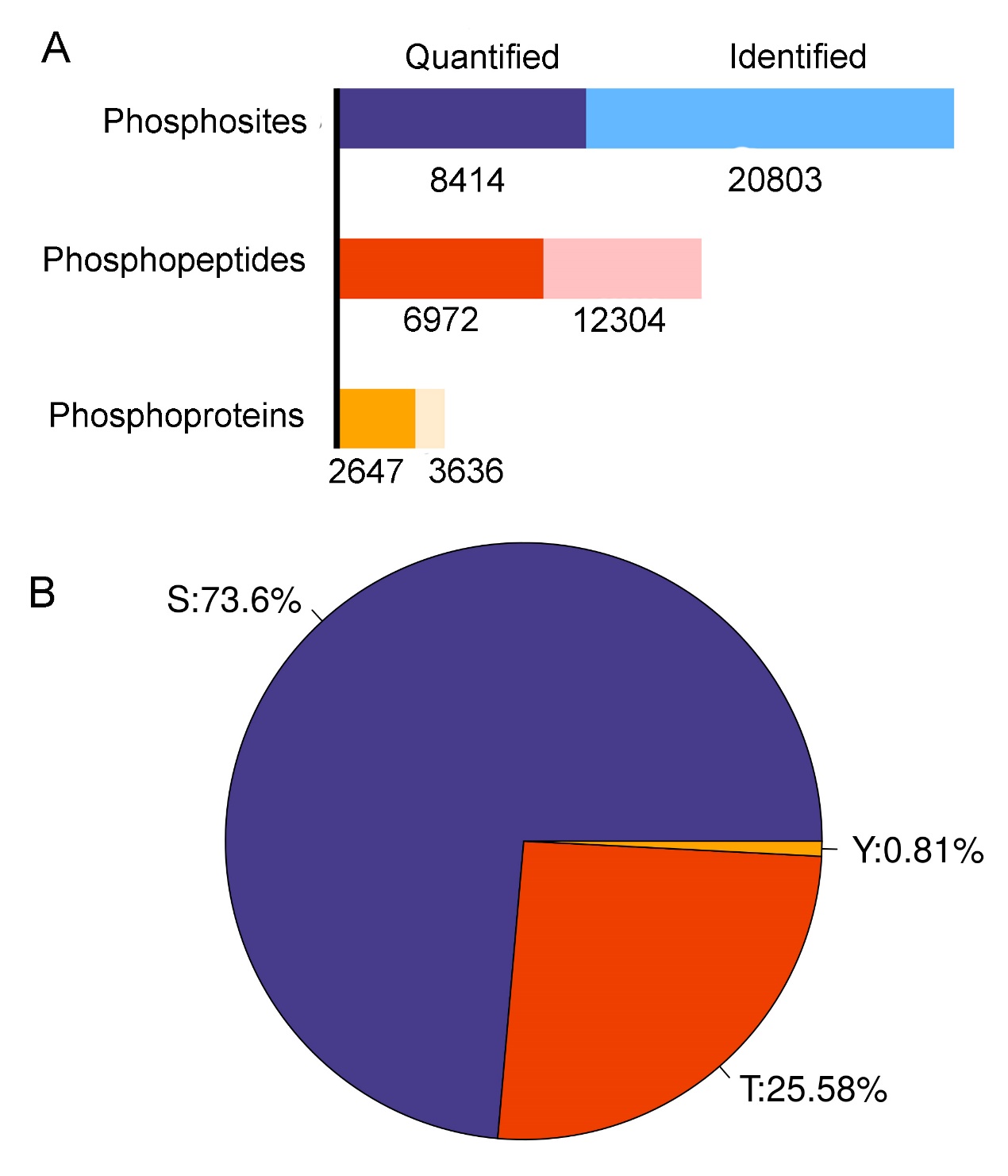


Fig.S7 Information on phosphorylation proteomics. A indicated the statistical histogram of identification and quantitative results; B indicated the analysis of the distribution of phosphorylation modification sites.


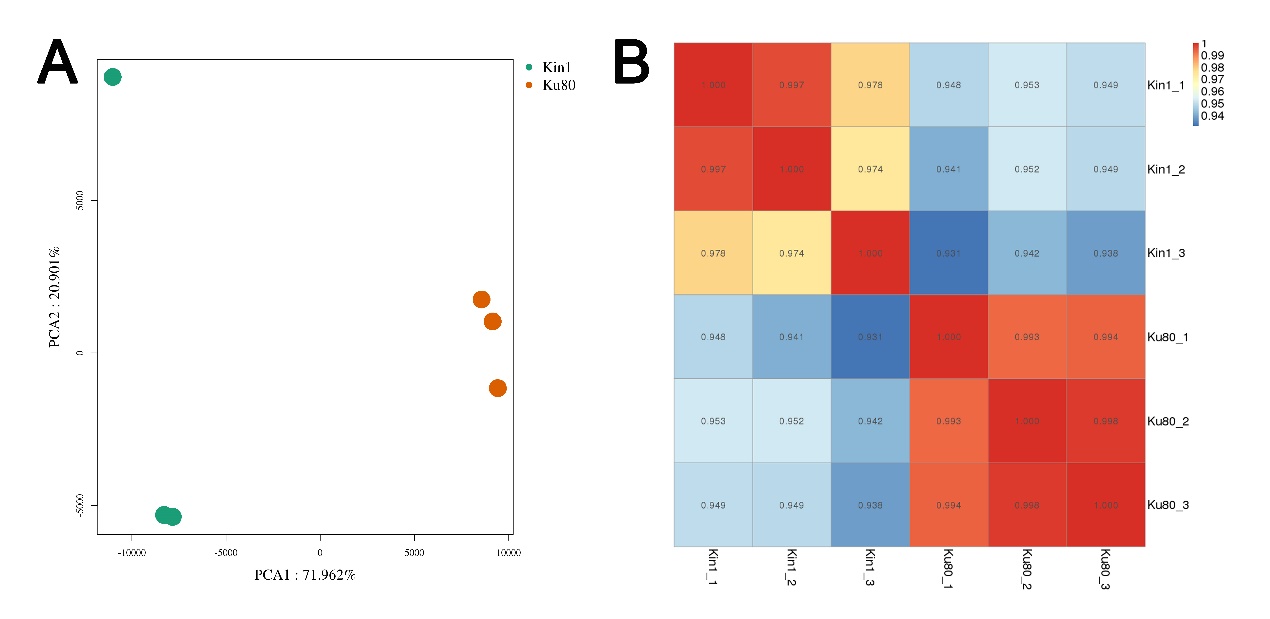


Fig.S8 Principal component analysis (PCA) (A) and heatmap (B) showed the gene expression in background strains Ku80 and *ΔMokin1* mutant.

Table.S1 Comprehensive analysis of proteomic data in MoKin1 deletion mutant.

Table.S2 Comprehensive analysis of phosphoproteomic data in MoKin1 deletion mutant.

Table.S3 Information of transcriptomic data in MoKin1 deletion mutant.

Table.S4 Comprehensive analysis of transcriptomic data in MoKin1 deletion mutant.

Table.S5 Homologous proteins of *S. cerevisiae* KIN1/KIN2 interacting proteins in *M. oryzae*

Table.S6 Comprehensive analysis of homologous proteins of *S. cerevisiae* KIN1/KIN2 interacting proteins in *M. oryzae*

Table.S7 Comprehensive analysis of MoKin1 pull down results
